# Sex-Related Differences in Prosthesis-Patient Mismatch Following Aortic Valve Replacement with Perceval Sutureless Valve

**DOI:** 10.3390/jcdd13020071

**Published:** 2026-01-31

**Authors:** Ali Aljalloud, Yusuf Shieba, Rashad Zayat, Ajay Moza, Ahmed Farghal Ahmed Mohammed

**Affiliations:** 1 Department of Cardiac Surgery, RWTH Aachen University Hospital, 52074 Aachen, Germany; rzayat@ukaachen.de (R.Z.); amoza@ukaachen.de (A.M.); amohammed@ukaachen.de (A.F.A.M.); 2 Department of Cardiothoracic Surgery, Faculty of Medicine, Qena University, Qena 83523, Egypt; yusuf.shieba@med.svu.edu.eg

**Keywords:** sex differences, aortic valve stenosis, prosthesis-patient mismatch, Perceval sutureless valve, aortic valve replacement, effective orifice area index

## Abstract

(1) Background: Prosthesis–patient mismatch (PPM) after aortic valve replacement (AVR) impairs left ventricular (LV) recovery and is more common in women due to smaller aortic dimensions. Although the Perceval sutureless valve provides larger effective orifice areas, sex-specific PPM outcomes remain unclear. This study evaluated sex-related differences in PPM incidence, severity, and early impact after Perceval AVR. (2) Methods: We retrospectively analyzed 139 patients (68 males, 71 females) who underwent Perceval AVR between 2016 and 2020. PPM was defined per Valve Academic Research Consortium-3 (VARC-3) criteria using indexed effective orifice area (EOAi) and stratified by body-mass-index (BMI) (<30 vs. ≥30 kg/m^2^). Echocardiography assessed hemodynamic performance. (3) Results: PPM was markedly more frequent in women than men (74.6% vs. 22.1%, *p* < 0.001). Among non-obese patients, 47.9% of females versus 16.2% of males developed PPM (*p* < 0.001). Women received smaller valves and consistently exhibited lower EOAi despite similar gradients. Postoperatively, females had reduced EOAi (0.8 vs. 0.9 cm^2^/m^2^, *p* < 0.001) but higher LV ejection fraction (55.8% vs. 49.5%, *p* = 0.004). Early clinical outcomes were comparable between sexes. (4) Conclusions: Despite favorable hemodynamics of sutureless AVR, anatomical sex differences result in persistently higher PPM rates in women, predominantly of moderate severity. Tailored strategies—including aortic root enlargement and sex-specific EOAi thresholds—may improve prosthesis selection and outcomes in female patients.

## 1. Introduction

Aortic stenosis (AS) is the most common form of valvular heart disease in the developed world, with a prevalence that rises significantly with age. For patients with severe, symptomatic AS, the prognosis is poor without intervention, making aortic valve replacement (AVR) the definitive, life-saving treatment. The patient population for AVR is often elderly with multiple comorbidities, which has driven the development of less invasive technologies to reduce operative risk [1].

The Perceval sutureless bioprosthesis is a significant technological advancement in this field. Composed of bovine pericardial leaflets mounted on a self-expanding nitinol stent, it is implanted surgically without the need for time-consuming annular sutures. This design shortens critical operative times and, by eliminating the internal sewing ring found in conventional valves, provides a larger effective orifice area (EOA) for any given annulus size. This feature is crucial for optimizing postoperative hemodynamics [2].

Despite the efficacy of AVR, a significant complication post-surgery arises when the valve does not adequately correspond to the patient’s anatomical dimensions, referred to as prosthesis-patient mismatch (PPM). PPM occurs when the implanted valve is inadequately sized, resulting in significant pressure variations that might result in complications such as inadequate recovery of the left ventricle, increased likelihood of heart failure, and, most concerning, an elevated risk of long-term mortality [3].

Patient sex is a critical variable in cardiovascular outcomes. Women typically have smaller aortic root and annular dimensions than men, even after adjusting for body size. This anatomical difference places female patients at a significantly higher baseline risk of developing PPM following conventional AVR, creating a well-documented “sex gap” in the incidence of this complication [4].

The Perceval valve has been reported to have an excellent hemodynamic profile, making it an optimal choice for reducing the likelihood of PPM, particularly in women with smaller aortic annuli. Research indicates that the Perceval valve markedly decreases PPM rates in comparison to conventional valves. However, it is notable that limited research has been conducted on the implications of this discrepancy between men and women. The efficacy of this technology in bridging the sex gap remains uncertain [5].

This study aims to investigate the sex-related differences in the incidence, severity, and clinical impact of PPM following AVR with the Perceval sutureless bioprosthesis.

## 2. Materials and Methods

### 2.1. Study Design and Patient Population

A retrospective analysis was conducted of all consecutive patients screened for surgical aortic valve replacement (AVR) with the Perceval sutureless bioprosthesis (LivaNova, London, United Kingdom) at our institution between January 2016 and May 2020.

**Figure 1:**
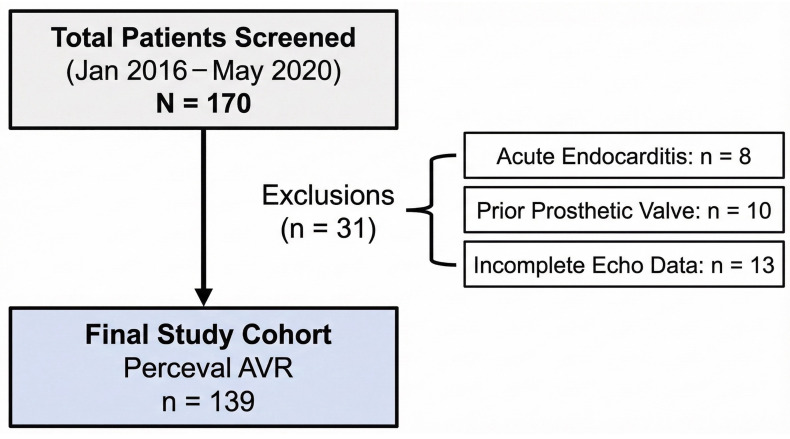
**STROBE flow diagram depicting patient selection.** AVR: aortic valve replacement; echo: echocardiography.

A total of 170 patients were initially assessed for eligibility. Of these, 31 patients were excluded based on the following criteria: active endocarditis (*n* = 8), prior prosthetic valve implantation (*n* = 10), or incomplete echocardiographic data preventing accurate calculation of EOAi (*n* = 13). The final study cohort comprised 139 patients (68 males, 71 females), who received either isolated or combined AVR procedures Figure 1. The investigation was approved by the local ethics committee (reference EK 151/09), and all patients provided written informed consent in accordance with the Declaration of Helsinki.

### 2.2. Surgical Technique

All procedures were performed by experienced cardiac surgeons through either a full median sternotomy or upper hemi-sternotomy, according to operative discretion and patient anatomy. After the establishment of cardiopulmonary bypass (CPB) and cardioplegic arrest, the native calcified aortic valve was excised, and annular debridement was completed. Valve sizing followed the manufacturer’s recommendations using dedicated sizers. The Perceval sutureless valve was then inserted in a collapsed state, positioned within the annulus, and deployed by balloon expansion to secure anchorage via its self-expanding nitinol stent. In combined procedures, coronary artery bypass grafting (CABG) or other concomitant cardiac interventions were performed as required. Standard de-airing and weaning from CPB were subsequently completed, and trans-esophageal echocardiography confirmed valve position and competence before chest closure.

### 2.3. Echocardiographic Assessment

Comprehensive transthoracic echocardiography (TTE) was performed pre-operatively and before hospital discharge by two independent, board-certified echocardiographers using commercially available ultrasound systems (GE Vivid E90, GE Vingmed Ultrasound, Horten, Norway). Measurements followed the current recommendations of the American Society of Echocardiography and European Association of Cardiovascular Imaging. The aortic valve area (AVA) was calculated by the continuity equation as AVA = CSA_LVOT_ × LVOT VTI/AV VTI. In the equation, CSA_LVOT_ represents the cross-sectional area of the left ventricular outflow tract derived from its diameter, LVOT VTI is the velocity-time integral of the LVOT flow obtained by pulsed-wave Doppler, and AV VTI is the velocity-time integral of the trans AV flow assessed using continuous-wave Doppler.

The Effective Orifice Area (EOA) of the prosthesis was calculated using the standard continuity equation: EOA = (CSA_LVOT_ × LVOT VTI)/prosthesis VTI, where VTI prosthesis is the velocity-time integral of the transprosthetic flow assessed using continuous-wave Doppler. The Indexed EOA (EOAi) was calculated by dividing the EOA by the patient’s Body Surface Area (BSA). Additional parameters included left-ventricular ejection fraction (LVEF), tricuspid annular plane systolic excursion (TAPSE), and assessment of paravalvular leakage (PVL) and aortic insufficiency (AI), graded as mild, moderate, or severe.

### 2.4. Definition of PPM

The presence and severity of PPM were determined according to the Valve Academic Research Consortium-3 (VARC-3) criteria [6].

No PPM: EOAi > 0.85 cm^2^/m^2^ for patients with BMI < 30 kg/m^2^, or >0.70 cm^2^/m^2^ for BMI ≥ 30 kg/m^2^.

Moderate PPM: EOAi 0.66–0.85 cm^2^/m^2^ (BMI < 30) or 0.56–0.70 cm^2^/m^2^ (BMI ≥ 30).

Severe PPM: EOAi ≤ 0.65 cm^2^/m^2^ (BMI < 30) or ≤ 0.55 cm^2^/m^2^ (BMI ≥ 30).

Analyses were performed separately for the non-obese (BMI < 30 kg/m^2^) and obese (BMI ≥ 30 kg/m^2^) subgroups to account for the BMI-specific EOAi thresholds.

### 2.5. Data Collection and Outcome Measures

Clinical, operative, and echocardiographic variables were retrieved from the institutional cardiac surgery database and electronic medical records. Recorded data included baseline demographics, comorbidities, operative details (CPB and cross-clamp times, concomitant procedures, valve size), and postoperative outcomes (ICU and hospital length of stay, need for pacemaker implantation, re-thoracotomy, neurologic events, and in-hospital mortality). Early hemodynamic performance of the prosthesis—peak pressure gradient (PPG), mean pressure gradient (MPG), and EOAi—was evaluated at discharge.

### 2.6. Statistical Analysis

Statistical analysis was performed using Stata 17.0 (StataCorp LLC, College Station, TX, USA) and Jamovi 2.6.3 (The Jamovi Project, Sydney, Australia). Continuous variables were expressed as mean ± standard deviation for normally distributed data or median (interquartile range) otherwise. Categorical variables were summarized as absolute numbers and percentages. Between-group comparisons (male vs. female) employed the independent-samples *t*-test or Mann–Whitney U-test for continuous variables and χ^2^ or Fisher’s exact test for categorical variables, as appropriate. Repeated measures variables were assessed using two-way ANOVA where applicable. When multiple comparisons were conducted (e.g., subgroup analyses of PPM severity by sex and BMI category), a post hoc pairwise analysis was performed using the false discovery rate (FDR) method to adjust for multiple testing. In addition to univariable comparisons, a multivariable logistic regression model was constructed to identify independent predictors of PPM. The dependent variable was the presence of any-degree PPM. Covariates were selected based on clinical relevance and baseline imbalance between sexes and included sex, body surface area, and prosthesis size category (S, M, L, XL). Adjusted odds ratios (OR) with 95% confidence intervals (CI) were reported. Model fit was assessed using the Hosmer–Lemeshow goodness-of-fit test. A two-sided *p*-value < 0.05 was considered statistically significant.

## 3. Results

A total of 139 patients (68 males [48.9%] and 71 females [51.1%]) underwent aortic valve replacement (AVR) with the Perceval sutureless prosthesis. Baseline pre-operative characteristics are summarized in Table 1. The two groups were well balanced with respect to age (76.6 ± 5.8 vs. 76.9 ± 5.3 years; *p* = 0.776), operative risk (EuroSCORE II: 2.0% (1.2, 4.0) vs. 2.9% (1.6, 4.9), *p* = 0.192), and BMI (28.4 (25.7, 30.5) vs. 27.3 (24.4, 30.8) kg/m^2^, *p* = 0.436). Males, however, had a significantly larger BSA (2.0 ± 0.2 vs. 1.8 ± 0.2 m^2^; *p* < 0.001) and a higher prevalence of diabetes mellitus (48.4% vs. 21.7%; *p* = 0.002). No other significant sex-based differences were observed in comorbidities or pre-operative valve morphology.

### 3.1. Operative and Early Postoperative Outcomes

Significant sex-based differences were observed in intra- and postoperative variables (Table 2). Females more frequently underwent isolated AVR (72.5% vs. 48.4%; *p* = 0.008), whereas males more often required concomitant CABG (45.3% vs. 24.6%; *p* = 0.020). Reflecting anatomical differences, prosthesis size distribution varied markedly: females more often received small and medium valves (25.4% and 35.2%), while males predominantly received extra-large valves (36.8% vs. 2.8%; *p* < 0.001). Males had longer aortic cross-clamp times (76.0 vs. 65.7 min; *p* = 0.024). Postoperative platelet counts (*p* = 0.058) and ICU length of stay (4.0 (2.0, 8.0) days vs. 3.0 (2.0, 5.0) days; *p* = 0.537) and length of hospital stay (15.0 (12.8, 24.2) days vs. 15.0 (12.0, 25.0) days; *p* = 0.702) were comparable between groups. At discharge, females exhibited a smaller indexed effective orifice area (EOAi = 0.8 (0.7, 0.9) vs. 0.9 (0.9, 1.0) cm^2^/m^2^; *p* < 0.001) but a higher left-ventricular ejection fraction (55% (55.0, 60.0) vs. 50% (45.0, 55.0), *p* = 0.014). Rates of postoperative complications and hospital stay duration were comparable between sexes.

### 3.2. Sex Differences in PPM

**Figure 2:**
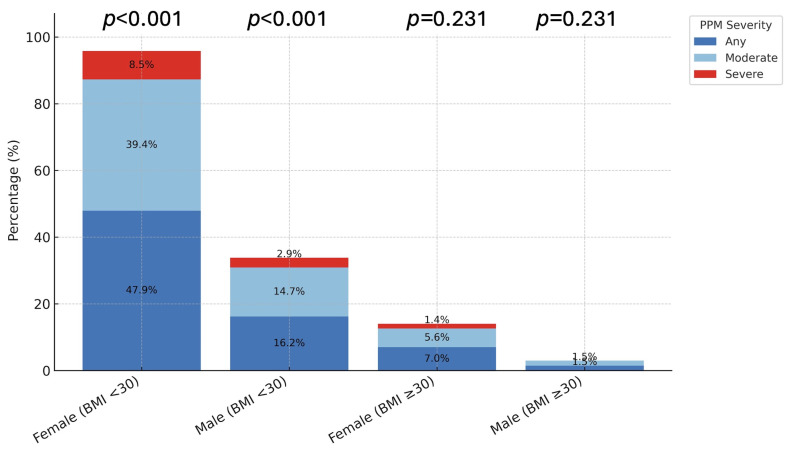
**Distribution of PPM by Sex and BMI Group.** The bar charts illustrate the prevalence of prosthesis-patient mismatch (PPM) stratified by Sex and Body Mass Index (BMI). (Left Panel) In non-obese patients (BMI < 30 kg/m^2^), females exhibited significantly higher rates of any (72.9%) and moderate (39.4%) PPM compared to males (*p* < 0.001). (Right Panel) In obese patients (BMI ≥ 30 kg/m^2^), the overall incidence of PPM was lower, and no significant sex differences were observed (*p* > 0.05). *p*-values indicate comparisons between sexes within each category.

Overall, PPM occurred significantly more often in females: 74.6% (*n* = 53) vs. 22.1% (*n* = 15) in males (*p* < 0.001; Table 3). This disparity was driven by patients with BMI < 30 kg/m^2^, in whom females exhibited markedly higher rates of both any PPM (47.9% vs. 16.2%; *p* < 0.001) and moderate PPM (39.4% vs. 14.7%; *p* < 0.001). Severe PPM was numerically higher in females (8.5% vs. 2.9%) but not statistically significant (*p* = 0.303). In obese patients (BMI ≥ 30 kg/m^2^), the overall incidence of PPM was low, with no significant sex-based differences (*p* > 0.1; Figure 2).

### 3.3. Valve Size and Hemodynamic Performance

A detailed comparison according to implanted valve size (Table 4) revealed a clear sex-related pattern. Size S prostheses were almost exclusively implanted in females (94.7%), whereas size XL valves were predominantly used in males (92.6%). Given the extremely small sample sizes in these subgroups (e.g., only *n* = one male with size S and *n* = two females with size XL), statistical comparisons for these specific categories lack sufficient power and should be interpreted with caution. Across the medium (M) and large (L) valve categories, males had larger BSA 2.0 ± 0.2 m^2^ vs. 1.8 ± 0.2 m^2^; *p* = 0.005 and *p* < 0.001, respectively, whereas BMI did not differ. Postoperative hemodynamic demonstrated superior EOAi values in males for three valve sizes: Size M (0.83 (0.80, 0.86) cm^2^/m^2^ vs. 0.80 (0.73, 0.83) cm^2^/m^2^; *p* = 0.023), size L (0.94 (0.91, 0.96) vs. 0.82 (0.80, 0.90) cm^2^/m^2^; *p* = 0.004), and size XL (1.0 (0.90, 1.1) cm^2^/m^2^ vs. 0.80 (0.70, 0.80) cm^2^/m^2^; *p* = 0.033). No significant sex differences were noted for EOAi in size S valves (*p* = 0.708) or for mean and peak gradients in any size group.

### 3.4. BMI-Stratified PPM Analysis

Among patients with BMI < 30 kg/m^2^ (*n* = 94), 48 (52.2%) had no PPM, 38 (40.4%) moderate PPM, and 8 (8.5%) severe PPM. Patients with PPM had significantly lower BSA (1.9 to 1.8 to 1.7 m^2^, *p* = 0.004) and BMI (25.9 to 25.7 to 23.4 kg/m^2^; *p* = 0.038). The distribution of PPM differed strongly by sex (*p* < 0.001; Table 5): 72.4% of females developed some degree of PPM (59.6% moderate, 12.8% severe) versus only 22.2% of males (17.8% moderate, 4.4% severe).

In patients with BMI ≥ 30 kg/m^2^ (*n* = 45), BSA and BMI were comparable across PPM strata (BSA *p* = 0.150; BMI *p* = 0.866). Overall, 37 (82.2%) had no PPM, 7 (15.6%) moderate, and 1 (2.2%) severe PPM. While the overall sex distribution trended toward significance in the unadjusted global test (*p* = 0.18; Table 6), women remained disproportionately represented among those with moderate-to-severe PPM (89% and 100%, respectively).

### 3.5. Post Hoc Analysis with FDR Correction

**Figure 3:**
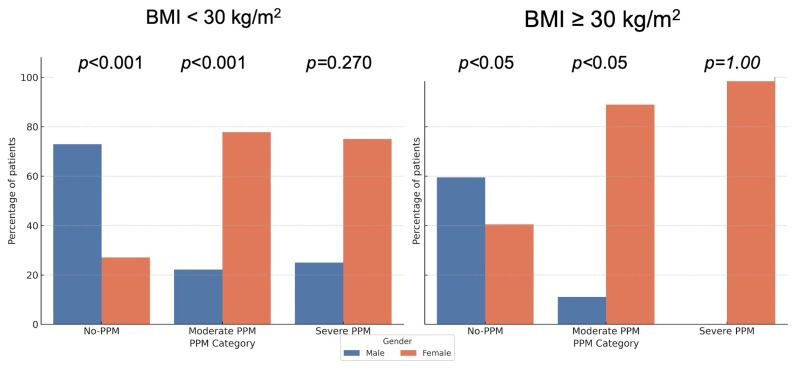
**Distribution of PPM by Sex with Post hoc *p*-values (FDR adjusted).** The bar charts display the prevalence of prosthesis-patient mismatch (PPM) stratified by Sex and Body Mass Index (BMI). *p*-values indicate pairwise comparisons between sexes within each category, adjusted for multiple testing using the False Discovery Rate (FDR) method. Significant sex differences persist in the non-obese group (BMI < 30 kg/m^2^), whereas differences in the obese group vary by severity category after adjustment.

To account for multiple testing, post hoc pairwise comparisons between males and females within each PPM severity category were performed using the false discovery rate (FDR) correction. Significant sex differences persisted for the non-obese subgroup in both the no-PPM and moderate PPM categories (*FDR-adjusted p* < 0.001), and for the obese subgroup in the no-PPM and moderate PPM categories (*FDR-adjusted p* < 0.05), while severe PPM remained non-significant (*p* = 1.00). These adjusted results are depicted in Figure 3.

### 3.6. Multivariable Analysis of Predictors for PPM

**Figure 4:**
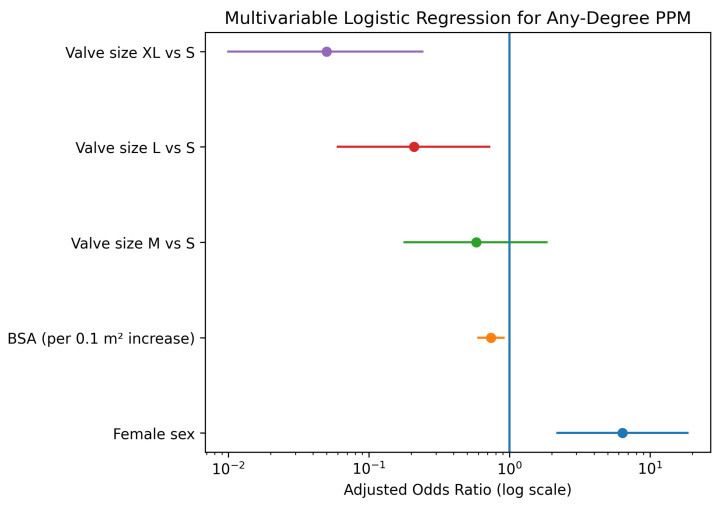
Forest plot displaying the results of the multivariable logistic regression analysis for predictors of prosthesis-patient mismatch. Purple indicates valve size XL vs. S, red valve size L vs. S, green valve size M vs. S, orange body surface area (per 0.1 m^2^ increase), and blue female sex. The vertical line represents an odds ratio of 1.

To determine whether the observed sex differences were independent of anatomical confounders, a multivariable logistic regression analysis was performed, adjusting for sex, BSA, and prosthesis size. In this adjusted model, female sex emerged as a strong, independent predictor of PPM (Adjusted Odds Ratio [OR]: 6.38, 95% Confidence Interval [CI]: 2.20–18.49; *p* < 0.001), Figure 4 and Table 7. Conversely, larger BSA was associated with a protective effect (OR per 0.1 m^2^ increase: 0.74, 95% CI: 0.60–0.91; *p* = 0.004). Regarding valve dimensions, implantation of larger prostheses significantly reduced the risk of PPM compared to size S, with the strongest risk reduction observed for size XL (OR: 0.05, 95% CI: 0.01–0.24; *p* < 0.001).

The plot illustrates the Adjusted Odds Ratios (OR) with 95% Confidence Intervals (CI). Female sex is a significant independent predictor of PPM, while higher Body Surface Area (BSA) and larger valve sizes (L and XL compared to S) are associated with a significantly lower risk.

## 4. Discussion

### 4.1. Summary of Main Findings

This study investigated sex-related differences in PPM following AVR with the Perceval sutureless bioprosthesis in 139 consecutive patients. The findings showed a significant difference in PPM incidence between female and male patients. PPM was observed significantly more often in females (74.6%) than in males (22.1%, *p* < 0.001). This difference was more evident among non-obese patients (BMI < 30 kg/m^2^), where 47.9% of females developed any degree of PPM, as opposed to 16.2% of males (*p* < 0.001). Females consistently received smaller prosthesis sizes and reached lower EOAi across all valve sizes (M, L, and XL). However, transvalvular gradients were comparable between sexes, and the majority of PPM cases in women were classified as moderate rather than severe. Importantly, early postoperative clinical outcomes—including ICU and hospital length of stay—were not adversely affected by this hemodynamic difference.

### 4.2. Comparison with Previous Studies

Our observation of a higher PPM incidence in women is consistent with a well-described “Sex-related disparity” in PPM after conventional AVR, the major cause of which relates to the fact that women have smaller aortic annuli and BSA, thus requiring a smaller prosthesis size [3]. Nevertheless, the overall PPM rates in our cohort, particularly the low rate of severe PPM (8.5% in women, 2.9% in men), compare favorably to historical rates associated with conventional sutured valves, often reporting higher rates of severe PPM [7]. The Perceval valve, by eliminating the internal sewing ring, is designed to maximize the effective orifice area, a feature that has been shown to reduce the overall incidence of PPM compared to stented bioprostheses [5]. Our findings support the valve’s benefit in reducing the most severe forms of PPM, but they also highlight that this advantage is not sufficient to fully equalize the risk between sexes. A recent large-scale study on PPM after surgical AVR, which included all valve types, similarly found that the incidence of any-degree PPM was significantly higher in women (31.9%) than in men (19.7%), further validating the persistent sex-based risk disparity observed in our Perceval-specific cohort [4].

### 4.3. Explanation of Possible Mechanisms

The persistent sex-related disparity in PPM after AVR is rooted in both anatomical and physiological differences. Women in our study received smaller prosthesis sizes (S and M) far more frequently than men, who predominantly received larger valves (XL), reflecting the smaller aortic root dimensions typical of female patients [8]. Despite the supra-annular design and favorable hemodynamics of the Perceval valve, the absolute valve size remains the primary determinant of EOA. When indexed to BSA, the smaller absolute EOA in women results in a lower EOAi, thus increasing the probability of PPM.

Beyond anatomical constraints, sex-specific myocardial and vascular adaptations further contribute to the higher PPM burden observed in women. Women with aortic stenosis typically develop a more concentric pattern of LV hypertrophy with smaller ventricular cavities, higher relative wall thickness, and preserved LVEF, a phenotype associated with heightened afterload sensitivity even when systolic function appears normal [9,10,11]. In addition, women exhibit lower systemic arterial and aortic root compliance, limiting annular distensibility and reducing the potential to accommodate larger prostheses during implantation [10,12]. Together, these myocardial and vascular characteristics amplify the hemodynamic impact of even modest reductions in EOAi and help explain why PPM remains disproportionately prevalent in female patients.

Finally, indexing-related hemodynamic factors may also magnify the observed sex gap. Current VARC-3 EOAi thresholds do not fully account for physiological differences between men and women [6]. Women typically have lower stroke volumes, smaller ventricular cavities, and higher relative wall thickness, all of which can artifactually depress EOAi after indexation and exaggerate the apparent severity of PPM [11,13,14]. Emerging evidence suggests that sex-specific or body-size–adjusted EOAi cutoffs may more accurately reflect true hemodynamic burden [15]. This issue is particularly relevant in our cohort, where most PPM cases in women were classified as moderate rather than severe, raising the possibility that current EOAi criteria may overestimate functional obstruction in small-stature female patients [15,16].

### 4.4. Clinical Implications

The high prevalence of moderate PPM in women—despite use of a supra-annular sutureless prosthesis—underscores the need for individualized surgical planning. Since our analysis was limited to the in-hospital period, we did not observe adverse clinical sequelae directly associated with PPM. However, preserved LVEF in women may mask increased afterload [9,17], and prior longitudinal data suggest that the adverse impact of PPM often emerges later, particularly in patients with low-flow physiology or impaired LV reserve [18,19]. These findings emphasize the importance of incorporating projected EOAi into preoperative assessment, especially in women with small annuli [11,20,21]. When predicted EOAi approaches the PPM threshold, strategies such as aortic root enlargement or selection of prostheses with ultra-high EOAi should be considered [22,23]. Given the short procedural time of the Perceval valve, root enlargement remains a feasible adjunct [24,25]. Enhanced postoperative surveillance may be warranted for women with PPM despite preserved LVEF, as EOAi may more accurately reflect hemodynamic load in this population [15,18,26].

### 4.5. Strengths and Limitations

The main strength of our work is the exclusive use of a single, advanced valve type, the Perceval, and the detailed stratification of the PPM incidence by sex and BMI according to the contemporary VARC-3 criteria [6]. This design enabled a precise evaluation of the ability of the Perceval valve to overcome the known sex-related challenge in PPM. Limitations of this study include its retrospective design. Although intraoperative annular sizing is routinely performed to guide prosthesis selection, these specific anatomical measurements were not consistently recorded in the study database, preventing a direct statistical correlation between native anatomy and valve size. The relatively small sample size (*n* = 139), particularly within the obese subgroup (BMI ≥ 30 kg/m^2^), limits the statistical power. Consequently, the lack of significant sex differences observed in obese patients should be interpreted with caution, as it may reflect limited power rather than true equivalence. Finally, postoperative echocardiography solely at discharge cannot fully capture the long-term hemodynamic performance of the valve, which may be further modulated by changes in cardiac output and LV function over time. Additionally, our study relied solely on resting transthoracic echocardiography. We did not perform pharmacological or exercise stress echocardiography, which could have provided further insights into dynamic valve performance and EOAi reserve under loading conditions. Furthermore, PPM was defined based exclusively on postoperatively measured EOAi to reflect the actual in vivo hemodynamic performance. We did not include an analysis comparing these measured values with projected EOAi based on reference charts, which could provide additional insights for preoperative planning in future studies.

## 5. Conclusions

Women undergoing AVR with the Perceval bioprosthesis demonstrated a substantially higher incidence of PPM, largely driven by smaller annular dimensions, sex-specific remodeling patterns, and EOAi indexation effects that may overestimate obstruction in small-stature patients. Most PPM in women was moderate, highlighting the need for individualized preoperative planning incorporating projected EOAi and, when borderline values are anticipated, consideration of aortic root enlargement or prostheses with higher effective orifice areas. Further studies with long-term follow-up are required to determine whether this hemodynamic disparity translates into survival differences and if sex-specific strategies can improve long-term patient survival.

## Figures and Tables

**Table 1:** Baseline characteristics of patients who underwent aortic valve replacement stratified by sex.

Variable	Male (*n* = 68)	Female (*n* = 71)	Total (*n* = 139)	*p*
Total *n* (%)	68 (48.9)	71 (51.1)	139	
Age (years)	76.6 (5.8)	76.9 (5.3)	76.7 (5.6)	*0.776*
EuroSCORE II (%)	2.0 (1.2, 4.0)	2.9 (1.6, 4.9)	2.3 (1.4, 4.6)	0.192
EuroSCORE I (%)	5.4 (3.2, 8.8)	4.7 (2.9, 7.3)	5.1 (3.0, 7.9)	0.628
BMI (kg/m^2^)	28.4 (25.7, 30.5)	27.3 (24.4, 30.8)	27.7 (25.1, 30.8)	0.436
BSA (m^2^)	2.0 (0.2)	1.8 (0.2)	1.9 (0.2)	<0.001 *
NYHA				0.146
Class I *n* (%)	4 (6.5)	0 (0.0)	4 (3.2)	
Class II *n* (%)	47 (75.8)	53 (82.8)	100 (79.4)	
Class III *n* (%)	11 (17.7)	10 (15.6)	21 (16.7)	
Class IV *n* (%)	0 (0.0)	1 (1.6)	1 (0.8)	
Preoperative Comorbidities				
Dyslipidemia *n* (%)	26 (40.6)	26 (37.7)	52 (39.1)	0.865
CKD *n* (%)	8 (12.3)	9 (13.0)	17 (12.7)	1.000
Ischemic stroke *n* (%)	7 (10.8)	7 (10.1)	14 (10.4)	1.000
COPD *n* (%)	6 (9.2)	8 (11.6)	14 (10.4)	0.869
Diabetes mellitus *n* (%)	31 (48.4)	15 (21.7)	46 (34.6)	0.002 *
PAD *n* (%)	5 (7.7)	5 (7.2)	10 (7.5)	1.000
AF *n* (%)	22 (33.8)	16 (23.2)	38 (28.4)	0.240
Platelet count (10^9^/L)	256.5 (116.9)	239.5 (71.4)	247.6 (95.8)	0.318
LDH (U/L)	209.0 (183.0, 246.5)	226.0 (190.5, 255.5)	217.0 (186.0, 252.0)	0.402
AS				0.531
Mild *n* (%)	1 (1.6)	0 (0.0)	1 (0.8)	
Moderate *n* (%)	3 (4.7)	2 (3.1)	5 (3.9)	
Severe *n* (%)	60 (93.8)	63 (96.9)	123 (95.3)	
AR				0.839
Mild *n* (%)	38 (59.4)	38 (58.5)	76 (58.9)	
Moderate *n* (%)	21 (32.8)	20 (30.8)	41 (31.8)	
Severe *n* (%)	5 (7.8)	7 (10.8)	12 (9.3)	
AVA (cm^2^)	0.80 (0.70, 0.90)	0.70 (0.60, 0.80)	0.73 (0.60, 0.90)	0.152

Data are presented as mean ± SD for normally distributed continuous variables, median (25th–75th percentile) for non-normally distributed variables, and number (percentage) for categorical variables. Comparisons were performed using Student’s t-test, Mann–Whitney U test, or Chi-square test as appropriate. * p < 0.05. Abbreviations: BMI, body mass index; BSA, body surface area; NYHA, New York Heart Association; CKD, chronic kidney disease; COPD, chronic obstructive pulmonary disease; PAD, peripheral artery disease; AF, atrial fibrillation; AS, aortic stenosis; AR, aortic regurgitation; AVA, aortic valve area.

**Table 2:** Sex-based differences in intraoperative, postoperative, and echocardiographic outcomes following aortic valve replacement with the Perceval sutureless bioprosthesis.

Variable	Male (*n* = 68)	Female (*n* = 71)	Total (*n* = 139)	*p*
Intraoperative Data				
Isolated AVR *n* (%)	31 (48.4)	50 (72.5)	81 (60.9)	0.008 *
AVR + CABG *n* (%)	29 (45.3)	17 (24.6)	46 (34.6)	0.020 *
AVR + MVR *n* (%)	1 (1.6)	0 (0.0)	1 (0.8)	0.970
Double Valve + CABG *n* (%)	0 (0.0)	1 (1.4)	1 (0.8)	1.000
Valve Size S *n* (%)	1 (1.5)	18 (25.4)	19 (13.7)	<0.001 *
Valve Size M *n* (%)	11 (16.2)	25 (35.2)	36 (25.9)	0.018 *
Valve Size L *n* (%)	31 (45.6)	26 (36.6)	57 (41.0)	0.367
Valve Size XL *n* (%)	25 (36.8)	2 (2.8)	27 (19.4)	<0.001 *
CPB time (minutes)	117.0 (42.3)	103.3 (40.9)	110.0 (42.0)	0.065
Cross Clamp (minutes)	76.0 (25.0)	65.7 (26.4)	70.7 (26.2)	0.024 *
Postoperative Data				
Platelet count (10^9^/L)	160.0 (107.8, 209.0)	132.0 (97.5, 175.5)	143.0 (99.5, 196.0)	0.058
LDH (U/L)	430.4 (117.6)	444.5 (115.4)	437.8 (116.2)	0.486
PM Implantation *n* (%)	5 (7.8)	5 (7.4)	10 (7.6)	1.000
AF *n* (%)	15 (23.4)	18 (26.5)	33 (25.0)	0.841
Re-thoracotomy *n* (%)	8 (12.5)	2 (2.9)	10 (7.6)	0.081
Pneumothorax *n* (%)	10 (15.6)	7 (10.3)	17 (12.9)	0.513
Delirium *n* (%)	12 (18.8)	7 (10.3)	19 (14.4)	0.256
Ischemic stroke *n* (%)	2 (3.1)	1 (1.5)	3 (2.3)	0.958
ICU Stay (days)	4.0 (2.0, 8.0)	3.0 (2.0, 5.0)	3.0 (2.0, 6.0)	0.537
LOS (days)	15.0 (12.8, 24.2)	15.0 (12.0, 25.0)	15.0 (12.0, 25.0)	0.702
Postoperative ECHO Data				
Mild PVL *n* (%)	4 (7.0)	6 (9.1)	10 (8.1)	0.929
Moderate PVL *n* (%)	0 (0.0)	1 (1.5)	1 (0.8)	1.000
Mild AI *n* (%)	5 (8.8)	10 (15.4)	15 (12.3)	0.405
Mean PG (mmHg)	12.2 (9.0, 17.0)	12.0 (10.0, 16.0)	12.0 (10.0, 16.8)	0.888
Peak PG (mmHg)	24.0 (17.2, 29.0)	23.0 (20.0, 29.8)	23.0 (20.0, 29.7)	0.625
EOAi (cm^2^/m^2^)	0.9 (0.9, 1.0)	0.8 (0.7, 0.9)	0.9 (0.7, 0.9)	<0.001 *
EF (%)	50.0 (45.0, 55.0)	55.0 (55.0, 60.0)	55.0 (50.0, 60.0)	0.014 *
TAPSE (mm)	14.0 (3.4)	15.0 (2.9)	14.5 (3.2)	0.163

Data are presented as mean ± SD for normally distributed continuous variables, median (25th–75th percentile) for non-normally distributed variables, and number (percentage) for categorical variables. * p < 0.05. Abbreviations: AF, atrial fibrillation; AI, Aortic valve insufficiency; AVR, aortic valve replacement; CABG, coronary artery bypass grafting; MVR, mitral valve replacement; CPB, cardiopulmonary bypass; ICU, intensive care unit; LDH, Laktatdehydrogenase; LOS, length of stay; PVL, paravalvular leak; PG, pressure gradient; EOAi, indexed effective orifice area; EF, ejection fraction; TAPSE, tricuspid annular plane systolic excursion.

**Table 3:** Distribution of PPM according to VARC between males and females.

Variable	Male (*n* = 68)	Female (*n* = 71)	Total (*n* = 139)	*p*
BMI < 30 (kg/m^2^)				
Any degree PPM *n* (%)	11 (16.2)	34 (47.9)	45 (32.4)	<0.001
Moderate PPM *n* (%)	10 (14.7)	28 (39.4)	38 (27.3)	<0.001
Severe PPM *n* (%)	2 (2.9)	6 (8.5)	8 (5.8)	0.303
BMI ≥ 30 (kg/m^2^)				
Any degree *n* (%)	1 (1.5)	5 (7.0)	6 (4.3)	0.100
Moderate PPM *n* (%)	1 (1.5)	4 (5.6)	5 (3.6)	0.190
Severe PPM *n* (%)	0 (0.0)	1 (1.4)	1 (0.7)	0.490

*PPM: patients-prosthesis mismatch, BMI: body mass index.*

**Table 4:** Comparison between females and males categorized according to valve size.

Valves Size S				
Variable	Male	Female	Total	*p*
Total *n* (%)	1 (5.3)	18 (94.7)	19	
BMI (kg/m^2^)	28.4 (NA)	28.7 (4.2)	28.7 (4.1)	0.934
BSA (m^2^)	1.9 (NA)	1.7 (0.2)	1.8 (0.2)	0.260
MPG (mmHg)	24.0 (24.0, 24.0)	12.0 (11.0, 15.6)	12.5 (11.0, 15.9)	0.171 *^†^*
PPG (mmHg)	48.0 (48.0, 48.0)	23.0 (22.0, 28.5)	23.0 (22.2, 28.9)	0.103 *^†^*
EOAi (cm^2^/m^2^)	0.83 (0.80, 0.86)	0.80 (0.73, 0.83)	0.80 (0.73, 0.86)	0.708 *^†^*
Valve size M				
Total *n* (%)	11 (30.6)	25 (69.4)	36	
BMI (kg/m^2^)	29.8 (5.9)	27.7 (5.6)	28.3 (5.7)	0.317
BSA (m^2^)	2.0 (0.2)	1.8 (0.2)	1.9 (0.2)	0.005 *
MPG (mmHg)	12.3 (11.0, 25.0)	12.0 (10.0, 19.0)	12.2 (10.2, 19.4)	0.459
PPG (mmHg)	23.0 (20.1, 40.0)	24.0 (21.0, 29.5)	23.5 (21.0, 37.2)	0.843
EOAi (cm^2^/m^2^)	0.90 (0.80, 0.90)	0.76 (0.73, 0.78)	0.76 (0.73, 0.90)	0.023
Valve size L				
Total *n* (%)	31 (54.4)	26 (45.6)	57	
BMI (kg/m^2^)	28.5 (4.5)	27.5 (4.6)	28.1 (4.5)	0.394
BSA (m^2^)	2.0 (0.2)	1.8 (0.2)	1.9 (0.2)	<0.001 *
MPG (mmHg)	12.2 (9.3, 17.0)	11.4 (10.0, 15.5)	12.0 (10.0, 16.0)	0.735
PPG (mmHg)	24.0 (17.0, 29.0)	22.2 (20.0, 29.4)	23.0 (19.0, 29.0)	0.525
EOAi (cm^2^/m^2^)	0.94 (0.91, 0.96)	0.82 (0.80, 0.90)	0.91 (0.80, 0.96)	0.004
Valve Size XL				
Total *n* (%)	25 (92.6)	2 (7.4)	27	
BMI (kg/m^2^)	28.2 (4.1)	30.9 (3.3)	28.4 (4.1)	0.365
BSA (m^2^)	2.0 (0.3)	2.0 (0.2)	2.0 (0.2)	0.976
MPG (mmHg)	11.6 (9.0, 14.6)	23.5 (20.3, 26.8)	12.4 (9.0, 17.1)	0.080 *^†^*
PPG (mmHg)	23.5 (19.5, 28.1)	39.3 (36.0, 42.7)	24.2 (20.5, 33.0)	0.095 *^†^*
EOAi (cm^2^/m^2^)	1.0 (0.90, 1.1)	0.80 (0.70, 0.80)	1.0 (0.9, 1.0)	0.033 *^†^*

^†^ Statistical comparisons in this subgroup are limited by extremely small sample size (n < 5) and should be interpreted with caution; * indicate statistical significance. Data are presented as mean ± SD or median (25th–75th percentile). Abbreviations: BMI, body mass index; BSA, body surface area; MPG, mean pressure gradient; PPG, peak pressure gradient; EOAi, indexed effective orifice area.

**Table 5:** Distribution of PPM according to the VARC definition and between males and females for patients with BMI < 30 kg/m^2^.

Patients with BMI < 30 (kg/m^2^)						
Variable		No-PPM	Moderate PPM	Severe PPM	Total	*p*
Total *n* (%)		48 (51.1)	38 (40.4)	8 (8.5)	94	
BSA (m^2^)	Mean (SD)	1.9 (0.2)	1.8 (0.2)	1.7 (0.1)	1.8 (0.2)	0.004 *
BMI (kg/m^2^)	Mean (SD)	25.9 (2.5)	25.7 (2.6)	23.4 (2.0)	25.6 (2.6)	0.038 *
Sex	Male *n* (%)	35 (72.9)	10 (26.3)	2 (25.0)	47 (50)	<0.001 *
	Female *n* (%)	13 (27.1)	28 (73.6)	6 (75.0)	47 (50)	

Data are presented as mean ± SD, median (25th–75th percentile), or number (percentage). * *p* < 0.05. Abbreviations: BMI, body mass index; BSA, body surface area; PPM, prosthesis-patient mismatch.

**Table 6:** Distribution of PPM according to the VARC definition and between males and females for patients with BMI ≥ 30 kg/m^2^.

Patients with BMI ≥ 30 (kg/m^2^)						
Variable		No-PPM	Moderate PPM	Severe PPM	Total	*p*
Total *n* (%)		39 (86.7)	5 (11.1)	1 (2.2)	45	
BSA (m^2^)	Mean (SD)	2.1 (0.2)	2.0 (0.2)	2.0 (NA)	2.1 (0.2)	0.150
BMI (kg/m^2^)	Mean (SD)	33.6 (3.3)	33.3 (3.7)	32.0 (NA)	33.5 (3.3)	0.866
Sex	Male *n* (%)	22 (56.4)	1 (20)	0 (0.0)	23 (48.9)	0.180
	Female *n* (%)	17 (43.6)	4 (80)	1 (100.0)	22 (51.1)	

*Data are presented as mean ± SD, median (25th–75th percentile), or number (percentage). Abbreviations: BMI, body mass index; BSA, body surface area; PPM, prosthesis-patient mismatch.*

**Table 7:** Multivariable logistic regression for any degree PPM. Independent predictors of prosthesis-patient mismatch (PPM) in the study cohort (*n* = 139). Adjusted Odds Ratios (OR) and 95% Confidence Intervals (CI) are reported. Abbreviations: BSA, body surface area; CI, confidence interval; OR, odds ratio; PPM, prosthesis-patient mismatch.

Variable	Adjusted OR	95% CI	*p*-Value
**Female sex**	6.38	2.20–18.49	<0.001
**BSA (per 0.1 m^2^ increase)**	0.74	0.60–0.91	0.004
**Valve size M vs. S**	0.58	0.18–1.84	0.36
**Valve size L vs. S**	0.21	0.06–0.72	0.013
**Valve size XL vs. S**	0.05	0.01–0.24	<0.001

## Data Availability

The data presented in this study are available from the corresponding author on reasonable request.

## Abbreviations

The following abbreviations are used in this manuscript:

AF	Atrial fibrillation
AI	Aortic insufficiency
AS	Aortic stenosis
AVA	Aortic valve area
AVR	Aortic valve replacement
BSA	Body surface area
BMI	Body mass index
CABG	Coronary artery bypass grafting
CKD	Chronic kidney disease
CPB	Cardiopulmonary bypass
EF	Ejection fraction
EOA	Effective orifice area
EOAi	Indexed effective orifice area
FDR	False discovery rate
ICU	Intensive care unit
LDH	Lactate dehydrogenase
LOS	Length of hospital stay
LV	Left ventricle
LVEF	Left-ventricular ejection fraction
LVOT	Left ventricular outflow tract
M	Medium valve size (Perceval) (kept because used in tables)
MPG	Mean pressure gradient
MVR	Mitral valve replacement
NYHA	New York Heart Association
PAD	Peripheral artery disease
PG	Pressure gradient
PM	Pacemaker
PPG	Peak pressure gradient
PPM	Prosthesis–patient mismatch
PVL	Paravalvular leak
S	Small valve size (Perceval)
SD	Standard deviation
TAPSE	Tricuspid annular plane systolic excursion
TTE	Transthoracic echocardiography
VARC-3	Valve Academic Research Consortium-3
XL	Extra-large valve size (Perceval)

## References

[1] Joseph J., Naqvi S.Y., Giri J., Goldberg S. (2017). Aortic stenosis: Pathophysiology, diagnosis, and therapy. Am. J. Med..

[2] Meco M., Montisci A., Miceli A., Panisi P., Donatelli F., Cirri S., Ferrarini M., Lio A., Glauber M. (2018). Sutureless Perceval Aortic Valve Versus Conventional Stented Bioprostheses: Meta-Analysis of Postoperative and Midterm Results in Isolated Aortic Valve Replacement. J. Am. Heart Assoc..

[3] Sá M.P., Jacquemyn X., Van den Eynde J., Chu D., Serna-Gallegos D., Ebels T., Clavel M.-A., Pibarot P., Sultan I. (2024). Impact of Prosthesis-Patient Mismatch After Surgical Aortic Valve Replacement: Systematic Review and Meta-Analysis of Reconstructed Time-to-Event Data of 122 989 Patients with 592 952 Patient-Years. J. Am. Heart Assoc..

[4] Springhetti P., Abdoun K., Clavel M.-A. (2024). Sex differences in aortic stenosis: From the pathophysiology to the intervention, current challenges, and future perspectives. J. Clin. Med..

[5] Belluschi I., Moriggia S., Giacomini A., Del Forno B., Di Sanzo S., Blasio A., Scafuri A., Alfieri O. (2017). Can Perceval sutureless valve reduce the rate of patient-prosthesis mismatch?. Eur. J. Cardiothorac. Surg..

[6] Généreux P., Piazza N., Alu M.C., Nazif T., Hahn R.T., Pibarot P., Bax J.J., Leipsic J.A., Blanke P., VARC-3 WRITING COMMITTEE (2021). Valve Academic Research Consortium 3: Updated endpoint definitions for aortic valve clinical research. Eur. Heart J..

[7] Fallon J.M., DeSimone J.P., Brennan J.M., O’Brien S., Thibault D.P., DiScipio A.W., Pibarot P., Jacobs J.P., Malenka D.J. (2018). The Incidence and Consequence of Prosthesis-Patient Mismatch After Surgical Aortic Valve Replacement. Ann. Thorac. Surg..

[8] Mohty D., Dumesnil J.G., Echahidi N., Mathieu P., Dagenais F., Voisine P., Pibarot P. (2009). Impact of prosthesis-patient mismatch on long-term survival after aortic valve replacement: Influence of age, obesity, and left ventricular dysfunction. J. Am. Coll. Cardiol..

[9] Tastet L., Kwiecinski J., Pibarot P., Capoulade R., Everett R.J., Newby D.E., Shen M., Guzzetti E., Arsenault M., Bédard É. (2020). Sex-Related Differences in the Extent of Myocardial Fibrosis in Patients with Aortic Valve Stenosis. JACC Cardiovasc. Imaging.

[10] Masiero G., Paradies V., Franzone A., Bellini B., De Biase C., Karam N., Sanguineti F., Mamas M.A., Eltchaninoff H., Fraccaro C. (2022). Sex-Specific Considerations in Degenerative Aortic Stenosis for Female-Tailored Transfemoral Aortic Valve Implantation Management. J. Am. Heart Assoc..

[11] Dobson L.E., Fairbairn T.A., Musa T.A., Uddin A., Mundie C.A., Swoboda P.P., Ripley D.P., McDiarmid A.K., Erhayiem B., Garg P. (2016). Sex-related differences in left ventricular remodeling in severe aortic stenosis and reverse remodeling after aortic valve replacement: A cardiovascular magnetic resonance study. Am. Heart J..

[12] Bahlmann E., Cramariuc D., Saeed S., Chambers J.B., Nienaber C.A., Kuck K.-H., Lønnebakken M.T., Gerdts E. (2019). Low systemic arterial compliance is associated with increased cardiovascular morbidity and mortality in aortic valve stenosis. Heart.

[13] Chakravarty T., Makar M., Ahmad Y. (2021). Patient-Prosthesis Mismatch After SAVR and TAVR: The Importance of Comparing Apples with Apples. JACC Cardiovasc. Interv..

[14] Pibarot P., Dumesnil J.G. (2006). Prosthesis-patient mismatch: Definition, clinical impact, and prevention. Heart.

[15] Herrmann H.C., Pibarot P., Wu C., Hahn R.T., Tang G.H.L., Abbas A.E., Playford D., Ruel M., Jilaihawi H., Sathananthan J. (2022). Bioprosthetic Aortic Valve Hemodynamics: Definitions, Outcomes, and Evidence Gaps: JACC State-of-the-Art Review. J. Am. Coll. Cardiol..

[16] Herrmann H.C., Daneshvar S.A., Fonarow G.C., Stebbins A., Vemulapalli S., Desai N.D., Malenka D.J., Thourani V.H., Rymer J., Kosinski A.S. (2018). Prosthesis-Patient Mismatch in Patients Undergoing Transcatheter Aortic Valve Replacement: From the STS/ACC TVT Registry. J. Am. Coll. Cardiol..

[17] Capoulade R., Clavel M.-A., Le Ven F., Dahou A., Thébault C., Tastet L., Shen M., Arsenault M., Bédard É., Beaudoin J. (2017). Impact of left ventricular remodelling patterns on outcomes in patients with aortic stenosis. Eur. Heart J. Cardiovasc. Imaging.

[18] Mohty D., Boulogne C., Magne J., Pibarot P., Echahidi N., Cornu E., Dumesnil J., Laskar M., Virot P., Aboyans V. (2014). Prevalence and long-term outcome of aortic prosthesis-patient mismatch in patients with paradoxical low-flow severe aortic stenosis. Circulation.

[19] Dayan V., Vignolo G., Soca G., Paganini J.J., Brusich D., Pibarot P. (2016). Predictors and Outcomes of Prosthesis-Patient Mismatch After Aortic Valve Replacement. JACC Cardiovasc. Imaging.

[20] Hahn R.T., Pibarot P. (2024). Prosthesis-patient mismatch in transcatheter and surgical aortic valve replacement. Ann. Cardiothorac. Surg..

[21] Pibarot P., Dumesnil J.G. (2000). Hemodynamic and clinical impact of prosthesis-patient mismatch in the aortic valve position and its prevention. J. Am. Coll. Cardiol..

[22] Rocha R.V., Manlhiot C., Feindel C.M., Yau T.M., Mueller B., David T.E., Ouzounian M. (2018). Surgical enlargement of the aortic root does not increase the operative risk of aortic valve replacement. Circulation.

[23] Flynn C.D., Williams M.L., Chakos A., Hirst L., Muston B., Tian D.H. (2020). Sutureless valve and rapid deployment valves: A systematic review and meta-analysis of comparative studies. Ann. Cardiothorac. Surg..

[24] Shrestha M., Folliguet T.A., Pfeiffer S., Meuris B., Carrel T., Bechtel M., Flameng W.J., Fischlein T., Laborde F., Haverich A. (2014). Aortic valve replacement and concomitant procedures with the Perceval valve: Results of European trials. Ann. Thorac. Surg..

[25] Marasco S.F., Banham T., Gregory S.D., Vu T., Stephens A.F. (2024). Use of sutureless valve in aortic root enlargement. Heart Lung Circ..

[26] Treibel T.A., Kozor R., Fontana M., Torlasco C., Reant P., Badiani S., Espinoza M., Yap J., Diez J., Hughes A.D. (2018). Sex dimorphism in the myocardial response to aortic stenosis. JACC Cardiovasc. Imaging.

